# Functional and Phenotypic Characterization of B Cells in the Teleost Adipose Tissue

**DOI:** 10.3389/fimmu.2022.868551

**Published:** 2022-05-10

**Authors:** Rocío Simón, Alba Martín-Martín, Esther Morel, Patricia Díaz-Rosales, Carolina Tafalla

**Affiliations:** Animal Health Research Center, Instituto Nacional de Investigación y Tecnología Agraria y Alimentaria, Consejo Superior de Investigaciones Científicas (CISA-INIA-CSIC), Madrid, Spain

**Keywords:** teleost fish, rainbow trout, adipose tissue, B cells, peritoneal antigens, IgM

## Abstract

The immune response of the adipose tissue (AT) has been neglected in most animal models until investigations in human and mice linked obesity to chronic inflammation, highlighting the immune nature of this tissue. Despite this, in teleost fish, only a few studies have addressed the immune role of the AT. These studies have mostly focused on reporting transcriptional changes in the AT in response to diverse intraperitoneally delivered stimuli. Although the presence of B cells within the AT was also previously revealed, these cells have never been phenotypically or functionally characterized and this is what we have addressed in the current study. Initially, the B cell populations present in the rainbow trout (*Oncorhynchus mykiss*) AT were characterized in comparison to B cells from other sources. As occurs in other rainbow trout tissues, IgM^+^IgD^+^, IgM^+^IgD^-^ and IgD^+^IgM^-^ B cell subsets were identified in the AT. Interestingly, AT IgM^+^IgD^-^ B cells showed a transcriptional profile that agrees with that of cells that have committed to plasmablasts/plasma cells, being this profile much more pronounced towards a differentiation state than that of blood IgM^+^IgD^-^ B cells. Accordingly, the IgM-secreting capacity of AT B cells is significantly higher than that of blood B cells. Additionally, AT IgM^+^IgD^+^ B cells also showed specific phenotypic traits when compared to their counterparts in other tissues. Finally, we established how these B cell subsets responded when rainbow trout were intraperitoneally injected with a model antigen. Our results demonstrate that the AT hosts plasmablasts/plasma cells that secrete specific IgMs, as happens in the peritoneal cavity and systemic immune tissues. Although the presence of these antigen-specific IgM-secreting cells was more abundant in the peritoneal cavity, these specific differentiated B cells were detected in the AT for long time periods at levels similar to those of spleen and head kidney. Our results provide new evidence regarding the immune role of the teleost AT, indicating that it functions as a secondary lymphoid organ that promotes immunity to peritoneal antigens.

## Introduction

In mammals, a distinction between visceral or subcutaneous adipose tissue (AT) can be established based on anatomical location, metabolic activity and immune role. From an immunological point of view, the visceral AT is of particular interest, as it is associated to the peritoneal cavity, has a high metabolic activity and contains differentiated organized lymphocyte areas. The visceral AT in humans includes structures such as the gonadal fat pad, the omentum, and the intestinal mesentery (1). All these structures are considered part of an endocrine organ composed of adipocytes and immune cells such as macrophages, dendritic cells, NKT cells and different subsets of T and B cells (2). In this environment, adipocytes secrete a wide range of hormones and cytokines that regulate immune cells, both locally within the AT and systemically (3). At the same time, AT leukocytes secrete soluble factors that reciprocally influence the activity of adipocytes, establishing a complex feedback-sensitive metabolic network to maintain metabolic homeostasis and low-grade chronic inflammation (2, 4). However, in obesity, this balance is disrupted, and the quantitative and functional response of AT immune cells when the metabolic microenvironment changes due to excess lipids, eventually leads to the occurrence of different disorders such as chronic inflammation, metabolic diseases, autoimmune syndromes or cancer (5). This established link between obesity and chronic inflammation together with the increasing prevalence of obesity throughout the world has encouraged in the past years many studies regarding the characterization and regulation of the immune components of the AT. Nevertheless, a complete understanding of the immune role of the AT is still lacking in most animal species.

In mammalian visceral AT, although macrophages are the most abundant immune cell type, different subsets of B cells are also found in structures such as the omentum (2, 5). Mammalian B cells are classified as B1 or B2 cells based on specific phenotypic characteristics, origin, and requirement of T cell help for antibody production (6, 7). Thus, in brief, B1 cells are innate-like B cells responsible for the production of natural antibodies in homeostasis that upon pathogen encounter mount rapid antibody responses in the absence of T cell help (8). These cells differentiate to either memory B cells or plasmablasts/plasma cells, but these are usually unswitched and short-lived IgM-secreting cells (9). B2 cells, on the other hand, are conventional B cells that differentiate in secondary lymphoid tissues and undergo germinal center reactions, interacting with cognate T helper cells, thereby differentiating into switched memory B cells or long-lived plasma cells (10). Although in secondary lymphoid tissues B2 cells vastly outnumber B1 cells, the ratio of B1 to B2 cells is much higher in AT, being approximately 1:1, equivalent to that of the peritoneal cavity (5). Interestingly, the role that each of these AT B cell subsets has in regulating immunity is quite different. Thus, while B2 cells have been shown to promote inflammation through the secretion of pro-inflammatory cytokines and IgG, B1 cells inhibit inflammation through the production of natural IgM and IL-10 (5). In addition to its contribution to maintaining an immunological equilibrium in homeostasis, the omentum has been shown to collect antigens from the peritoneal cavity and support responses to these antigens. Although these include mostly T cell-independent responses orchestrated by local B1 cells (11, 12), additional studies have demonstrated that the omentum is able to mount T cell-dependent B cell responses, which involve isotype switching, somatic hypermutation, and some degree of affinity maturation, despite the lack of identifiable follicular dendritic cells (13).

In teleost fish, a well-developed visceral AT is present, in which several leukocyte types have been identified, including B and T cells (14–16). Nevertheless, an in-depth characterization of their immune elements has not yet been achieved nor its exact role in immunity defined. To date, most of the few studies that have addressed the immune role of the AT have been undertaken in rainbow trout (*Oncorhynchus mykiss*). These studies revealed that lymphocytes and particularly B cells seem to be the one of the most abundant immune cell types in the AT (14, 16). Additionally, they reported significant changes in the levels of transcription of a wide range of immune factors produced by the AT, such as pro-inflammatory molecules, cytokines or marker genes for different leukocyte subpopulations in response to diverse intraperitoneally-delivered stimuli (14, 16–18). In Atlantic salmon (*Salmo salar* L.), a recent study, also reported transcriptional changes in the AT after an intraperitoneal challenge with *Piscirickettsia salmonis*. The changes observed in the levels of transcription of marker genes for different adaptive cell types led the authors to hypothesize that the AT is a site for antigen presentation and B cell activation (19). In this context, it seemed adequate to thoroughly characterize the different B cell populations present in teleost AT and investigate their response to immune stimulation, and this is what we have undertaken in the current study using the rainbow trout as a model. We focused this study on IgM and/or IgD-expressing B cell subsets, given that the levels of transcription of IgT are much lower than those of IgM in the rainbow trout AT (14), and because IgT^+^ B cells constitute an independent B cell linage thought to be specialized in mucosal responses (20).

In rainbow trout, previous studies have identified three different B cell subsets based on their pattern of IgM/IgD surface expression. Thus, IgM^+^IgD^+^ B cells constitute the major B cell subset in systemic compartments and seem to correspond to naïve mature B cells (21). These fish B cells downregulate IgD after encountering antigen as mammalian B cells do (21, 22), once they start a differentiation program towards plasmablasts/plasma cells and become IgM^+^IgD^−^ B cells. Finally, IgD^+^IgM^−^ B cells have also been detected in fish species such as catfish (*Ictalurus punctatus*) (23), and in rainbow trout, where they were especially predominant in some specific mucosal tissues such as intestine or gills (24, 25). As in mammals, the precise role of this B cell subset is still lacking in teleosts (26). In the current study, we investigated the presence of these three B cell subsets in rainbow trout AT through flow cytometry and immunofluorescence analysis (IFA), confirming that in contrast to what occurs in systemic tissues, the IgM^+^IgD^-^ B cell subset is the most abundant in the AT in homeostasis. A phenotypic and functional characterization of these populations in comparison to B cells from other tissues revealed some specific traits of AT B cell subsets that are also discussed. Finally, we established how AT B cells respond to an intraperitoneal immunization with a model thymus-independent (TI) antigen, TNP-LPS, once again comparing the response to that of the peritoneal cavity and systemic immune organs. The results provide new insights on the immune nature of the teleost AT, pointing to this tissue as an active immune site with a relevant role in the responsiveness to peritoneal antigens.

## Materials and Methods

### Experimental Fish

Healthy rainbow trout (*Oncorhynchus mykiss*) of different sizes were obtained from Cifuentes fish farm (Guadalajara, Spain) and maintained at the animal facilities of the Animal Health Research Center (CISA-INIA-CSIC), in a recirculating water system at 15°C, with a 12:12 h light/dark photoperiod. Fish were fed twice a day with a commercial diet (Skretting). Before any experimental procedure, fish were acclimatized to laboratory conditions for two weeks, and during this period, no clinical signs were ever observed.

### Leukocyte Isolation

Rainbow trout of different sizes (approximately 10-15 cm or 20-25 cm, depending on the experiment) were sacrificed by benzocaine (Sigma) overdose to obtain total leukocyte populations from spleen, head kidney, peritoneal cavity, AT and peripheral blood. Cell suspensions from spleen and head kidney were obtained by pushing the tissues through 100 µm nylon cell strainers (BD Biosciences) using Leibovitz’s medium (L-15, Gibco) supplemented with 100 IU/ml penicillin, 100 µg/ml streptomycin (P/S), 10 U/ml heparin (Sigma-Aldrich) and 2% fetal calf serum (FCS, Gibco). Peritoneal leukocytes were obtained by injecting 2 ml of cold L-15 medium supplemented with P/S, 10 U/ml heparin and 2% FCS into the peritoneal cavity. After gently massaging the abdominal surface, the medium containing the peritoneal cells was harvested from the peritoneal cavity with a pipette tip inserted into a peritoneal incision. The injection and harvesting were repeated twice to collect any remaining fluid from the cavity. To isolate leukocytes from the AT, the tissue was removed from the peritoneal cavity (**Figure S1**) and initially washed in L-15 containing P/S and 5% FCS at 4°C for 30 min. Thereafter, the AT was incubated for 30 min with agitation at room temperature (RT) in calcium and magnesium-free Hanks Balanced Salt Solution 1X (HBSS, Gibco) supplemented with P/S, containing 1 mM ethylenediaminetetraacetic acid (EDTA, Thermo Fisher Scientific) and 1 mM DL-dithiothreitol (DTT, Sigma-Aldrich). After this incubation, leukocytes released into the medium were washed once to discard EDTA and DTT and resuspended in L-15 supplemented with P/S and 5% FCS. Subsequently, a tissue digestion using 2 mg/ml collagenase type IV from *Clostridium histolyticum* (Sigma-Aldrich) in L-15 supplemented with P/S was performed for 30 min at RT. Later, the digested AT tissues were collected, pushed through 100 μm nylon cell strainers and the resulting suspension mixed with the previously collected AT leukocytes. For all tissues, cell suspensions were placed onto 30/51% discontinuous Percoll (GE Healthcare) density gradients and centrifuged at 500 x *g* for 30 min at 4°C, without brake (14). To isolate leukocytes from peripheral blood, blood was diluted 10 times with L-15 medium containing P/S, 10 U/ml heparin and 5% FCS. Peripheral blood leukocytes (PBLs) were isolated placing diluted blood samples onto 51% Percoll (GE Healthcare) density gradients and centrifuged at 500 x *g* for 30 min at 4°C, without brake. For all tissues, cells at the interface were collected and washed in L-15 medium containing P/S and 5% FCS. The viable cell concentration was determined by trypan blue exclusion.

### *In Vivo* Stimulation

Rainbow trout of approximately 10-15 cm received 50 µg of 2,4,6-Trinitrophenyl hapten conjugated to lipopolysaccharide (TNP-LPS) (Biosearch technologies) in 200 µl of sterile saline solution (0.9% sodium chloride, ClNa) by means of an intraperitoneal injection. The conjugated LPS was the high molecular weight form of *Escherichia coli* with repeating polysaccharide O-chain. A mock-immunized group (control) received an intraperitoneal injection of 200 µl of sterile saline solution. Sampling was performed after 7, 14 and 28 days, collecting 8 rainbow trout from each group. After sacrificing the rainbow trout by benzocaine overdose, leukocytes were isolated from spleen, head kidney, peritoneal cavity, peripheral blood and AT as described above to quantify the number of total and TNP-specific trout IgM-secreting cells by ELISpot and to perform flow cytometry analysis of the IgM^+^ B cell population.

For immunofluorescence staining, the AT from 6 rainbow trout from each group were collected at day 21 post-immunization. The collected tissues were fixed in 4% paraformaldehyde for 24 h and processed for paraffin embedding following routine histological procedures, as previously described (27).

### Flow Cytometry Analysis

To characterize B cell populations in homeostasis, peritoneal, AT and blood leukocytes (2 x 10^5^ cells), obtained from unstimulated 20-25 cm rainbow trout were used. Cells were washed in staining buffer (PBS containing 1% FBS and 0.5% sodium azide) and co-incubated for 1 h at 4°C with anti-IgM (1.14) [mAb mouse IgG1 coupled to R-phycoerythrin (R-PE), 1 μg/ml], anti-MHC II β-chain [mAb mouse IgG1 coupled to fluorescein isothiocyanate (FITC), 2 μg/ml] and anti-IgD [mAb mouse IgG1 coupled to allophycocyanin (APC), 10 μg/ml]. All mAbs were specific for rainbow trout and had been previously characterized (28–30). After the incubation, cells were washed twice with staining buffer.

To undertake the cytometry analysis of B cells in the *in vivo* experiment, spleen, head kidney and blood leukocytes (2 x 10^5^ cells) obtained from fish intraperitoneally immunized with TNP-LPS or mock-immunized were incubated with anti-trout IgM coupled to R-PE (1 µg/ml) in staining buffer for 1 h at 4°C. In the case of the AT and peritoneal leukocytes, cells were simultaneously stained with anti-IgD conjugated with APC (10 μg/ml). Thereafter, cells were washed twice with staining buffer.

In all cases, cells were analyzed on a FACS Celesta™ flow cytometer (BD Biosciences) equipped with BD FACSDiva™ software and the flow cytometry analysis was performed with FlowJo^®^ v.10. (TreeStar). All the incubations were performed at 4°C. Isotype controls were also included in all cases, to verify the specific binding of the antibodies. During the setting up of the experiments, cell viability was checked using DAPI (0.2 μg/ml), and only live cells were included in the analysis. Cell viability was always higher than 95% in our experimental conditions.

### Enzyme-Linked ImmunoSpot Assay (ELISpot)

ELISpot was used to quantify the number of total or TNP-specific IgM secreting B cells. ELISpot plates containing Immobilon-P membranes (Millipore) were activated with 70% ethanol and coated with 2 µg/ml of an anti-trout IgM mAb or with 5 µg/ml of TNP-BSA overnight at 4°C in agitation. Non-specific binding sites were blocked by incubation with 2% bovine serum albumin (BSA, Sigma Aldrich) in phosphate buffered saline (PBS) for 2 h at RT. After that, leukocytes from naïve fish (5 x 10^3^ cells per well) or from fish immunized with TNP-LPS or mock-immunized (1 x 10^4^ cells per well) were added to the wells. After 24 h of incubation at 20°C, cells were washed 5 times with PBS and plates blocked with 2% BSA in PBS for 1 h at RT. After blocking, biotinylated anti-trout IgM was added to the plates at a concentration of 1 µg/ml and incubated for 1 h at RT. Following 5 additional washing steps in PBS, the plates were developed using 100 ng/ml of streptavidin-HRP for 1 h at RT, washed again 5 times with PBS and incubated with 3-amino 9-ethylcarbazole (Sigma-Aldrich) for 30 min at RT in the dark. The substrate reaction was stopped by washing the plates with tap water. Once the membranes were dried, the number of spots in each well was determined using an AID iSpot Reader System (Autoimmun Diagnostika GMBH). Different types of controls were included in all experiments (wells without cells, wells not coated with anti-IgM and wells in which no secondary antibody was added).

### Immunofluorescence and Confocal Microscopy

Leukocyte suspensions from the AT were collected as described above and seeded on poly-L-lysine (0.01% solution, Sigma)-coated slides (4 x 10^5^ cells per fish) and incubated at RT for 1 h in a humidified chamber. The slides were then fixed in 4% paraformaldehyde solution for 30 min at RT. The fixed samples were incubated for 1 h at RT with blocking solution (TBS, pH 7.5 containing 5% BSA and 0.5% saponin) to minimize non-specific adsorption of the antibodies to the coverslip. The samples were then incubated with mAbs against trout IgM (coupled to FITC, 17 μg/ml) and trout IgD (coupled to APC, 50 μg/ml) for 1 h at RT in a humidified chamber. Slides were counterstained with 1 μg/ml DAPI (Sigma-Aldrich) for 10 min at RT, rinsed with PBS 1x and mounted with Fluoromount (Sigma-Aldrich) for microscopy.

AT samples obtained from fish immunized with TNP-LPS or mock-immunized (at day 21 post-immunization) were fixed in 4% paraformaldehyde and processed for paraffin embedding as described before (27). Thereafter, 4 μm-thick tissue sections were mounted on Superfrost Plus slides (Menzel- Gläser). To determine the percentage of proliferating IgM^+^ B cells at the different conditions, a double immunofluorescence detection of IgM and the proliferating cell nuclear antigen (PCNA) was performed. For this, the mouse monoclonal antibody directed against IgM was combined with an antibody directed against PCNA, an intracellular molecule whose expression and synthesis is linked with cellular proliferation (31). First, antigen retrieval was performed by heating the slides in Tris–EDTA buffer (10 mM Tris base, 1 mM EDTA, pH 9) in a microwave oven for 5 min at 800 W and 5 min at 450 W. Thereafter, non-specific binding was blocked with 5% BSA in Tris-buffered saline (TBS). Tissues were then incubated with the primary antibody anti-trout-IgM (27) diluted 1:10. Incubation with a secondary anti-mouse IgG1 antibody conjugated with AlexaFluor^®^488 (ThermoFisher) was followed by a further incubation with a mouse IgG2 anti-PCNA antibody conjugated with AlexaFluor^®^647 (BioLegend) and counterstaining with DAPI (1 μg/ml, Sigma). All the incubation steps were performed for 1 h at RT in the dark. Tissue autofluorescence was finally blocked by incubation with 0.3% Sudan black B in 70% ethanol for 10 min. Sections were then rinsed with TBS and mounted with Fluoromount (Sigma-Aldrich) for microscopy.

Laser scanning confocal microscopy images were acquired with an inverted Zeiss Axiovert LSM 880 microscope with Zeiss Zen software. Images were analyzed and processed with Zeiss Zen and Adobe Photoshop CS6 software packages. In the case of the *in vivo* experiment, tissue images were analyzed in 10 digital fields (400x magnification) from each tissue in 6 different individuals from each experimental condition (control and TNP-LPS).

### Sorting of AT and Blood IgM^+^ B Cell Populations

AT and blood leukocytes obtained from non-stimulated rainbow trout of approximately 20-25 cm were stained with anti-trout IgM coupled to FITC (0.5 µg/ml) and anti-trout IgD coupled to APC (10 μg/ml) for 1 h at 4°C in the dark in staining buffer. Following several washing steps, cells were resuspended in staining buffer and IgM^+^IgD^+^ and IgM^+^IgD^-^ cells isolated by flow cytometry using a BD FACSAria III cell sorter (BD Biosciences) based on the fluorescence emitted by the anti-IgM and anti-IgD antibodies. Approximately 20,000 isolated IgM^+^IgD^+^ and IgM^+^IgD^-^ B cells were collected for subsequent RNA isolation and analysis of immune gene transcription using the Power SYBR Green Cells-to-Ct Kit as described below.

### Analysis of Gene Transcription in Sorted AT and Blood B Cell Populations

Total RNA was extracted from FACS isolated AT and blood IgM^+^IgD^-^ and IgM^+^IgD^+^ B cell populations using the Power SYBR Green Cells-to-Ct Kit (Invitrogen) following the manufacturer’s instructions. RNA was treated with DNase during the process to remove genomic DNA that might interfere with the PCR reactions. Reverse transcription was also performed using the Power SYBR Green Cells-to-Ct Kit following the manufacturer’s instructions. To evaluate the levels of transcription of the different genes, real time PCR was performed with a LightCycler 96 System instrument (Roche) using SYBR Green PCR core Reagents (Applied Biosystems) and specific primers previously described (**Table S1**). Samples obtained from individual fish were analyzed in duplicate under the following conditions: 10 min at 95°C, followed by 40 amplification cycles (15 s at 95°C and 1 min at 60°C). A melting curve for each primer set was obtained by reading fluorescence every degree between 60 and 95°C to ensure that only a single PCR product had been amplified. The expression of individual genes was normalized to the relative expression of the housekeeping gene elongation factor 1 α (EF-1α), and the expression levels were calculated using the 2-ΔCt method, where ΔCt is determined by subtracting the EF-1α value from the target Ct (Ct cut-off set to 38). EF-1α was selected as reference gene according to the MIQE guidelines (32), given that no statistical differences were detected among Ct values obtained for EF-1α in the different samples. Negative controls with no template and *minus* reverse transcriptase controls were included in all the experiments.

### Statistical Analysis

Data handling, statistical analyses, and graphic representation were performed using Microsoft Office Excel 2010 and GraphPad Prism version 7.02 (GraphPad Software). Statistical analyses were performed using a two-tailed Student´s *t* test and the differences between the mean values were considered significant on different degrees, where ∗ means p ≤ 0.05, ∗∗ means p ≤ 0.01, and ∗∗∗ means p ≤ 0.005.

## Results

### Analysis of B Cell Subpopulations in the Trout AT by Flow Cytometry

**Figure 1:**
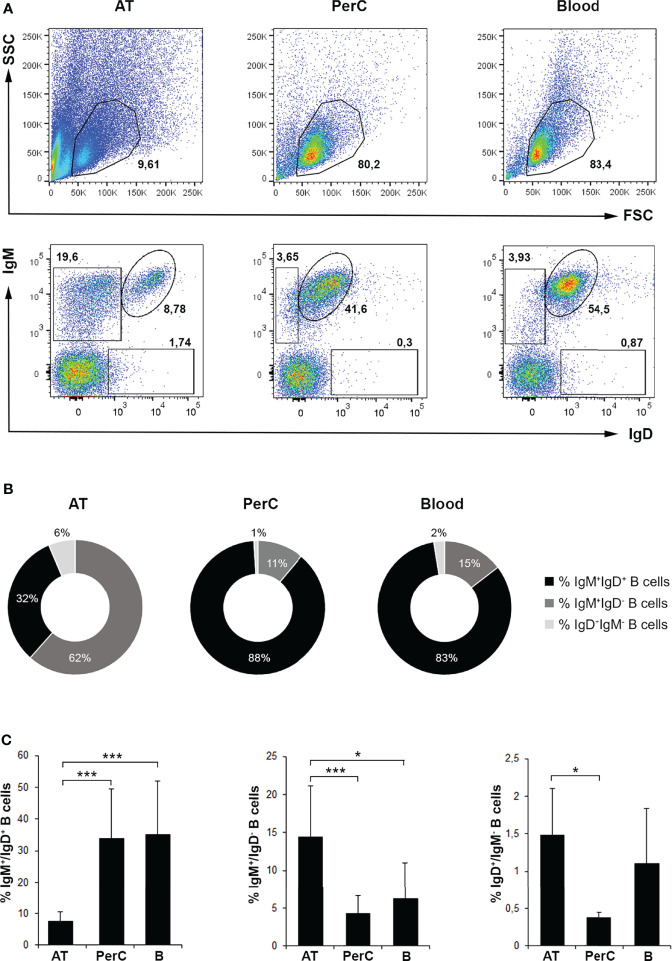
Flow cytometry analysis of B cell subsets in rainbow trout AT compared to peritoneal cavity (PerC) and blood leukocyte populations. Leukocytes isolated from AT, PerC and blood were labeled with mAbs against trout IgM and IgD and analyzed by flow cytometry. **(A)** Representative dot plots from each tissue are shown. Upper panels correspond to dot plots showing the forward scatter and side scatter (FSC/SSC) profile of isolated leukocytes in which the lymphoid gates were defined. Lower panels correspond to dot plots showing IgM and IgD fluorescence intensities defining the different B cell subpopulations in each tissue (IgM^+^IgD^+^; IgM^+^IgD^-^; IgD^+^IgM^-^). **(B)** Pie charts showing the ratio of the three different subpopulations of IgM/D-bearing B cells in each tissue. **(C)** Graphs showing the mean percentages of B cell subsets among total lymphoid cells in each tissue (mean + SD; *n* = 8 fish). AT, Adipose tissue; PerC, Peritoneal cavity; B, Blood. Asterisks denote significantly different values between indicated groups (*p ≤ 0.05 and ***p ≤ 0.005).

In the current study, we have characterized the different B cell populations present in the AT in homeostasis, comparing them to those of the peritoneal cavity and blood. In concordance with the previous study (14), IgM^+^IgD^+^, IgM^+^IgD^-^ and IgD^+^IgM^-^ B cell subsets were identified by flow cytometry in the AT, as occurs in the peritoneal cavity and blood, being IgM^+^IgD^-^ B cells the most abundant population in the AT (**Figures 1A–C**). Specifically, we found that IgM^+^IgD^-^ AT B cell population represented approximately 62% of all B lymphocytes expressing either IgM and/or IgD on the cell membrane and approximately 15% of all leukocytes, which is significantly higher than the percentage represented by this population in peritoneal cavity and blood (~11% and ~15% of all IgM and/or IgD B cells and ~4% and ~6% of all leukocytes, respectively) (**Figures 1A–C**). Both in peritoneal cavity and blood, the major lymphocyte population is constituted by IgM^+^IgD^+^ B cells, representing approximately 88% and 83% of all IgM and/or IgD B cells and 34% and 35% of all leukocytes, respectively. However, this subpopulation of B cells represents approximately 32% of all IgM and/or IgD B cells and only ~7.5% of all leukocytes in the AT (**Figures 1B, C**). Interestingly, we also found that IgD^+^IgM^-^ B cells are more abundant in AT compared to the other tissues analyzed, representing approximately ~6% of all IgM and/or IgD B cells and ~1,5% of all leukocytes in the AT (**Figures 1A–C**). This difference in the percentage of IgD^+^IgM^-^ B cells among tissues was only statistically significant when comparing AT to peritoneal cavity (**Figure 1C**).

**Figure 2:**
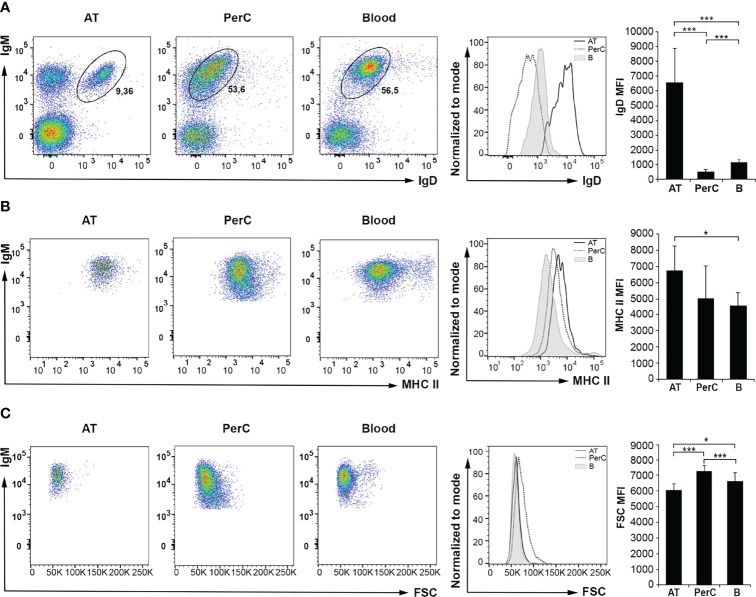
Flow cytometry analysis of IgD and MHC II surface expression levels and cell size of IgM^+^IgD^+^ B cells in rainbow trout AT compared to peritoneal cavity (PerC) and blood IgM^+^IgD^+^ B cells. Leukocytes isolated from AT, PerC and blood were labeled with mAbs against trout IgM, IgD and MHC II and analyzed by flow cytometry. **(A)** Representative dot plots with IgM and IgD **(A)** or MHC II **(B)** fluorescence intensities from each tissue are shown, along with an histogram with IgD **(A)** or MHC II **(B)** surface expression levels on IgM^+^IgD^+^ B cells from tissues of one representative fish, and the corresponding graph showing the mean fluorescence intensity (MFI) values (mean + SD; *n* = 8 fish). **(C)** Representative dot plots showing IgM and FSC values of IgM^+^IgD^+^ B cells in each tissue are shown, along with an histogram with FSC values of IgM^+^IgD^+^ B cells from one representative fish, and the corresponding graph showing FSC MFI values (mean + SD; *n* = 8 fish). AT, Adipose tissue; PerC, Peritoneal cavity; B, Blood. Asterisks denote significantly different values between indicated groups (*p ≤ 0.05 and ***p ≤ 0.005).

Next, we compared the phenotype of the IgM^+^IgD^+^ B cell subpopulation in the different tissues by analyzing in these cells the levels of IgD and major histocompatibility complex class II (MHC II) surface expression as well as their size (**Figures 2A–C**). As shown in the corresponding dot plots, histograms and graphs, the levels of surface IgD (IgD mean fluorescence intensity, MFI) were significantly higher in AT IgM^+^IgD^+^ B cells than those observed in blood or peritoneal IgM^+^IgD^+^ B cells (**Figure 2A**). In the case of MHC II, surface expression levels (MHC II MFI) were significantly more elevated in AT IgM^+^IgD^+^ B cells, although in this case, the levels of expression were only significantly higher when compared to those of the blood subpopulation but not when compared to peritoneal cells (**Figure 2B**). Finally, we also analyzed the size of IgM^+^IgD^+^ B cells (referred to as FSC MFI) in the three tissues. The results show that the largest B cells were those isolated from peritoneal cavity, followed by blood B cells and then AT B cells. The differences in cell size observed between the different groups of B cells from each tissue were, in all cases, statistically different. Thus, IgM^+^IgD^+^ AT B cells express the highest levels of both IgD and MHC II on their cell surface, whilst being smaller than the equivalent subpopulation in peritoneal cavity or blood.

### Comparative Analysis Between IgM^+^IgD^+^ and IgM^+^IgD^-^ AT B Cell Subpopulations by Flow Cytometry and Immunofluorescence

**Figure 3:**
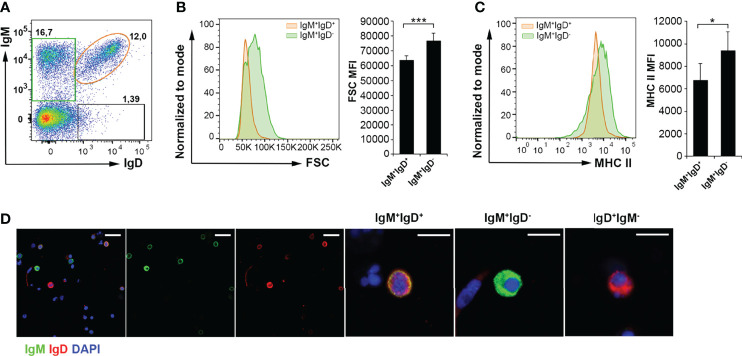
Characterization of IgM^+^ B cells subpopulations in rainbow trout AT by flow cytometry and confocal microscopy analysis. Rainbow trout AT leukocytes were labeled with anti-IgM, anti-IgD and anti-MHC-II trout mAbs. **(A)** Representative dot plot showing IgM and IgD profiles in AT samples is shown to define the different gated B cell subsets. **(B)** Histogram showing FSC values of IgM^+^IgD^+^ B cells (orange) compared to IgM^+^IgD^-^ B cells (green), from one representative fish, along with the corresponding graph showing the MFI values for FSC (mean + SD; *n* = 8 fish). Asterisks denote significantly different values between indicated groups (***p ≤ 0.005). **(C)** Histogram showing MHC II expression levels on the surface of IgM^+^IgD^+^ B cells (orange) compared to that of IgM^+^IgD^-^ B cells (green) in one representative fish is shown, along with the corresponding graph showing the MFI values for MHC II (mean + SD; *n* = 8 fish). Asterisks denote significantly different values between the indicated groups (*p ≤ 0.05). **(D)** Isolated AT leukocytes were labeled with anti-IgM (green) and anti-IgD (red) and counterstained with DAPI (blue) and the different subpopulations of AT B cells (IgM^+^IgD^+^; IgM^+^IgD^-^ and IgD^+^IgM^-^) visualized by confocal microscopy. Examples of each B cell subset are shown in higher magnification images. Scale bars in left-hand images: 20 μm; scale bars in right-hand images: 5 μm.

It has been previously established that as trout B cells differentiate towards a plasmablast/plasma cell profile, they lose surface IgD and they increase their size (20, 33). In the same way, an attenuated MHC II expression is viewed as a hallmark of plasmablast/plasma cell differentiation (33, 34). For this reason, we decided to analyze by flow cytometry both the size and the surface MHC II expression levels of the two major B cell populations in the trout AT, IgM^+^IgD^+^ and IgM^+^IgD^-^ cells. As expected, IgM^+^IgD^-^ AT B cells are larger than IgM^+^IgD^+^ cells (**Figures 3A, B**). However, the levels of surface MHC II expression were also significantly more elevated in IgM^+^IgD^-^ B cells (**Figures 3A, C**), in contrast to what *a priori* would be expected from a more differentiated cell. Nevertheless, an immunofluorescence analysis (IFA), confirmed that IgM^+^IgD^-^ B cells were more frequent in the AT compared to IgM^+^IgD^+^ and IgD^+^IgM^-^ B cells (**Figure 3D**), but also evidenced that IgM^+^IgD^-^ B cells, as well as IgD^+^IgM^-^ B cells, had a larger cytoplasm-to-nucleus ratio than IgM^+^IgD^+^ B cells (**Figure 3D**). The size comparison and MHC-II surface levels between IgM^+^IgD^+^ and IgM^+^IgD^-^ B cells subsets was also carried out in the peritoneal cavity and blood. In both tissues, IgM^+^IgD^-^ B cells were significantly larger than IgM^+^IgD^+^ cells (**Figure S2A, B**). However, no significant differences were found in MHC II surface expression levels between IgM^+^IgD^+^ and IgM^+^IgD^-^ B cells in these tissues (**Figure S2A, B**).

### Quantification of Total IgM-Secreting Cells in AT and Transcriptional Profile of Sorted IgM^+^ AT B Cells

**Figure 4:**
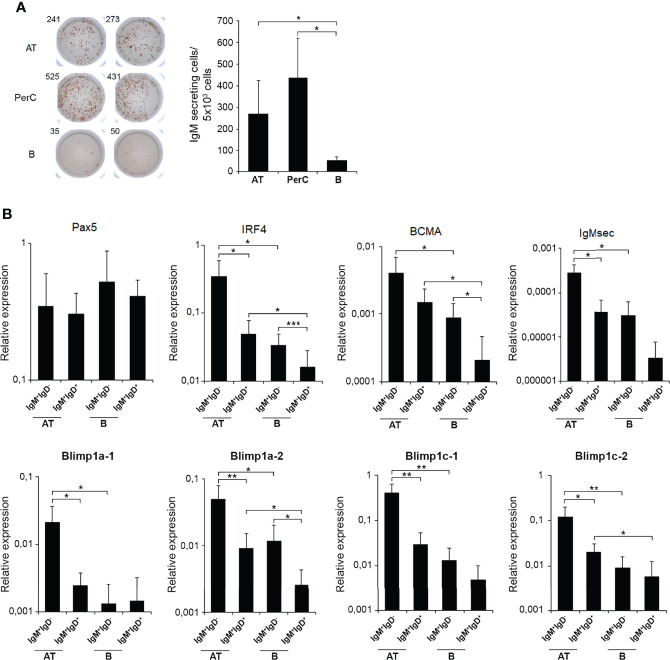
ELISPOT analysis of IgM-secreting cells in rainbow trout AT, peritoneal cavity and blood and transcriptional analysis of IgM^+^ sorted B cells from AT and blood. **(A)** Isolated leukocytes from rainbow trout AT, peritoneal cavity (PerC) and blood were plated in ELISPOT plates, previously coated with anti-IgM mAb for 24 h. After incubation, cells were washed away and a biotinylated anti-IgM mAb used to detect number of spot forming cells. Duplicates for a representative individual (left) in each tissue and quantification of spot forming cells (right) from 6 independent fish are shown (mean + SD). Asterisks denote significantly different values among groups as indicated (*p ≤ 0.05). **(B)** AT and blood IgM^+^IgD^+^ and IgM^+^IgD^-^ B cell subpopulations were FACS isolated and the transcription levels of different genes associated with B cell differentiation studied by real time PCR. Results are shown as relative expression values to endogenous control EF-1α (mean + SD; *n*=7 fish). Asterisks denote significantly different values between the indicated groups (*p ≤ 0.05; **p ≤ 0.01 and ***p ≤ 0.005).

We next investigated the IgM-secreting capacity of AT B cells in homeostasis compared to that of peritoneal and blood B cells, by means of an ELISPOT assay. The results obtained showed that the frequency of IgM-secreting cells in AT leukocyte cultures was significantly higher than in blood leukocytes, but not as high as within peritoneal leukocytes (**Figure 4A**). These results suggest that the AT contains a much higher proportion of B cells that are differentiated towards a plasmablast/plasma cell profile than blood in homeostasis, but not as high as that found in the peritoneal cavity.

Nevertheless, to further characterize phenotypically the AT B cell subsets, we decided to study the transcriptional profile of IgM^+^IgD^+^ and IgM^+^IgD^-^ B cell populations in AT and blood. In this experiment, peritoneal cells were not included as the number of cells isolated from this tissue is too small for sorting and posterior transcriptomic analysis. When we FACS isolated IgM^+^IgD^-^ B cells and IgM^+^IgD^+^ B cells from AT and blood leukocytes cultures, we found that AT IgM^+^IgD^-^ cells have a transcriptional profile that in fact corresponds to cells that have already committed to plasmablasts/plasma cells. Consequently, AT IgM^+^IgD^-^ cells have significantly higher levels of transcription of IRF4, secreted IgM and all Blimp1 homologues tested than those observed in the IgM^+^IgD^+^ AT subpopulation (**Figure 4B**). Interestingly, this transcriptional plasmablast-like profile was much more pronounced in AT IgM^+^IgD^-^ cells than that observed in blood IgM^+^IgD^-^ B cells, given that AT IgM^+^IgD^-^ cells had significantly higher levels of transcription of IRF4, BCMA, secreted IgM and the four Blimp1 homologues than their counterparts in blood (**Figure 4B**).

### Quantification of Total and TNP-Specific IgM-Secreting Cells in Intraperitoneally Immunized Fish

**Figure 5:**
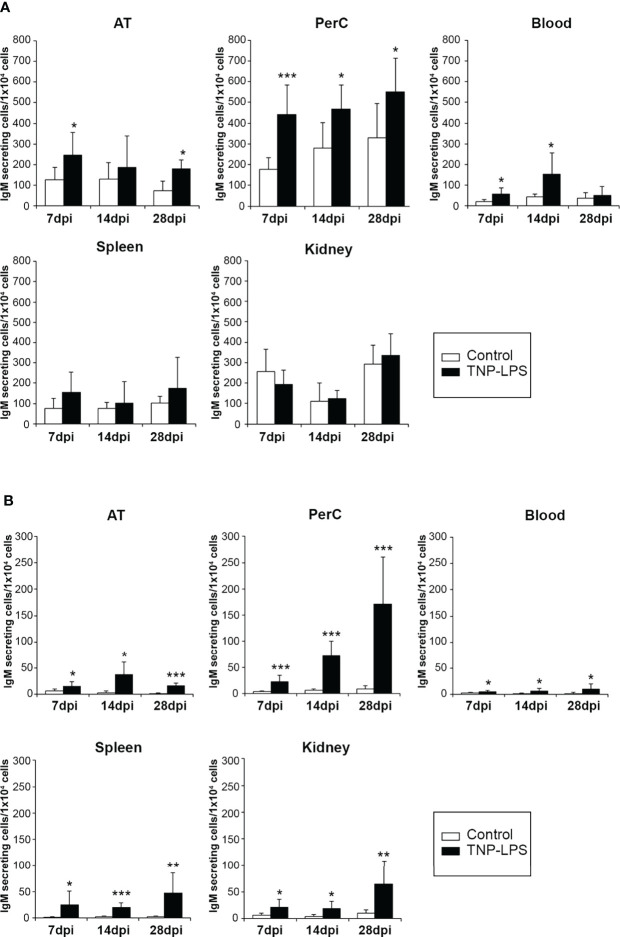
Quantification of total and TNP-specific IgM-secreting cells after the intraperitoneal immunization of rainbow trout with TNP-LPS. **(A)** The amount of total IgM-secreting cells in AT, peritoneal cavity (PerC), blood, spleen and head kidney (HK) was established by ELISPOT as described in the Materials and Methods section. Results are shown as mean number of IgM-secreting cells per 1 x 10^4^ total leukocytes + SD (n = 8). Asterisks denote significantly different values between groups as indicated (*p ≤ 0.05 and ***p ≤ 0.005). **(B)** To determine the amount of cells secreting TNP-specific IgMs, ELISPOT plates were coated with TNP-BSA and developed using a biotinylated anti-trout IgM mAb. Results are shown as mean number of IgM-secreting cells per 1 x 10^4^ cells + SD (n = 8). Asterisks denote significantly different values among groups as indicated (*p ≤ 0.05 **p ≤ 0.01 and ***p ≤ 0.005).

Having performed the characterization of AT B cell subsets in homeostasis, we next wanted to establish how these AT B cells respond to an antigen encounter. For this, rainbow trout were intraperitoneally injected with a model TI antigen, TNP-LPS, to which previous studies have established that rainbow trout are highly responsive (35, 36). At different times post-immunization (7, 14 and 28 days post-immunization), TNP-LPS-treated and control rainbow trout were sacrificed and the number of B cells secreting total or TNP-specific IgMs quantified through ELISPOT in the AT as well as in the peritoneal cavity, blood, spleen and head kidney. As shown in **Figure 5A**, the frequency of total IgM-secreting cells significantly increases along time in the AT, peritoneal cavity and blood of immunized fish when compared to control fish. Interestingly, in the case of spleen and head kidney, no statistically significant differences were observed between immunized fish and control fish at any time post-immunization (**Figure 5A**). However, when we studied the number of cells secreting TNP-specific IgMs, we found that in all cases, fish immunized with TNP-LPS have a significantly higher number of TNP-specific IgM-secreting cells than control fish, at all time points studied and in all tissues tested (**Figure 5B**). These differences were especially remarkable in the peritoneal cavity, where the number of B cells secreting TNP-specific IgMs increased along time (**Figure 5B**). Nevertheless, these B cells secreting TNP-specific IgMs were detected in the AT for long time periods at levels similar to those observed in central immune organs such as spleen or head kidney (**Figure 5B**).

### Flow Cytometry Analysis of IgM^+^ B Cells in Intraperitoneally Immunized Fish

**Figure 6:**
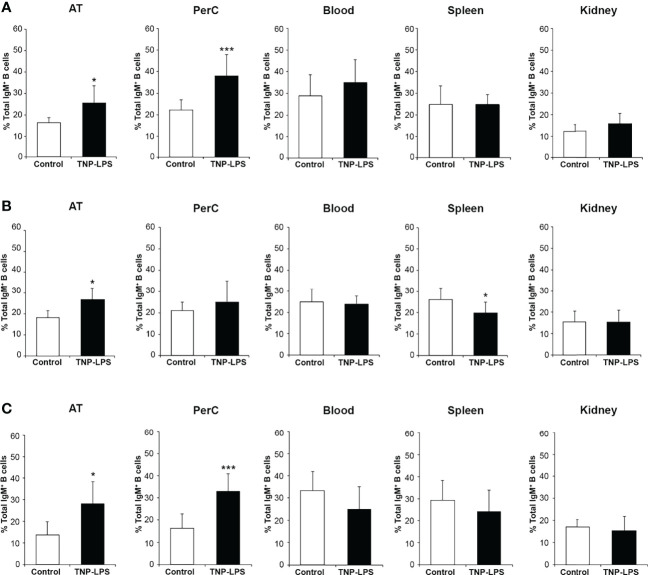
Percentage of total IgM^+^ B cells after the intraperitoneal immunization of rainbow trout with TNP-LPS. Rainbow trout were immunized intraperitoneally with TNP-LPS or mock-immunized (control) and leukocytes isolated at different times post-immunization from AT, peritoneal cavity (PerC), blood, spleen and head kidney (HK). Leukocytes were then incubated with an anti-trout IgM mAb conjugated to PE and analyzed by flow cytometry. Graphs shown the mean percentage of the total IgM^+^ B cells in each tissue at days 7 **(A)**, 14 **(B)** and 28 **(C)** post-immunization (mean + SD; *n* = 8). Asterisks denote significantly different values between the TNP-LPS immunized group and the control group (*p ≤ 0.05 and ***p ≤ 0.005). See **Figure S2**, where representative dot plots showing the percentage of total IgM^+^ B cells (IgM vs FSC) are included for each group and tissue at the three sampling times.

To further establish the contribution of AT B cells to peritoneal responses, we also evaluated by flow cytometry the percentage of total IgM^+^ B cells in the AT, peritoneal cavity, blood, spleen and head kidney of fish intraperitoneally immunized with TNP-LPS or mock-immunized at the different time points post-injection. At day 7 post-immunization, the percentage of total IgM^+^ B cells in the AT and peritoneal cavity was significantly higher in immunized fish than in control fish (**Figure 6A** and **Figure S3A**). In blood, spleen and head kidney, no differences in the percentage of total IgM^+^ B cells were found between groups at this time point. At day 14 post-immunization, the percentage of total IgM^+^ B cells was significantly higher in the immunized group compared to the control group only in the AT (**Figure 6B** and **Figure S3B**). At this point, in the spleen, the percentage of total IgM^+^ B cells significantly decreased in the immunized group in comparison to the control group (**Figures 6B** and **S3B**). After 28 days, we found again that the percentage of total IgM^+^ B cells in AT and peritoneal cavity from fish immunized with the antigen was significantly higher than that of control fish (**Figures 6C** and **S3C**).

**Figure 7:**
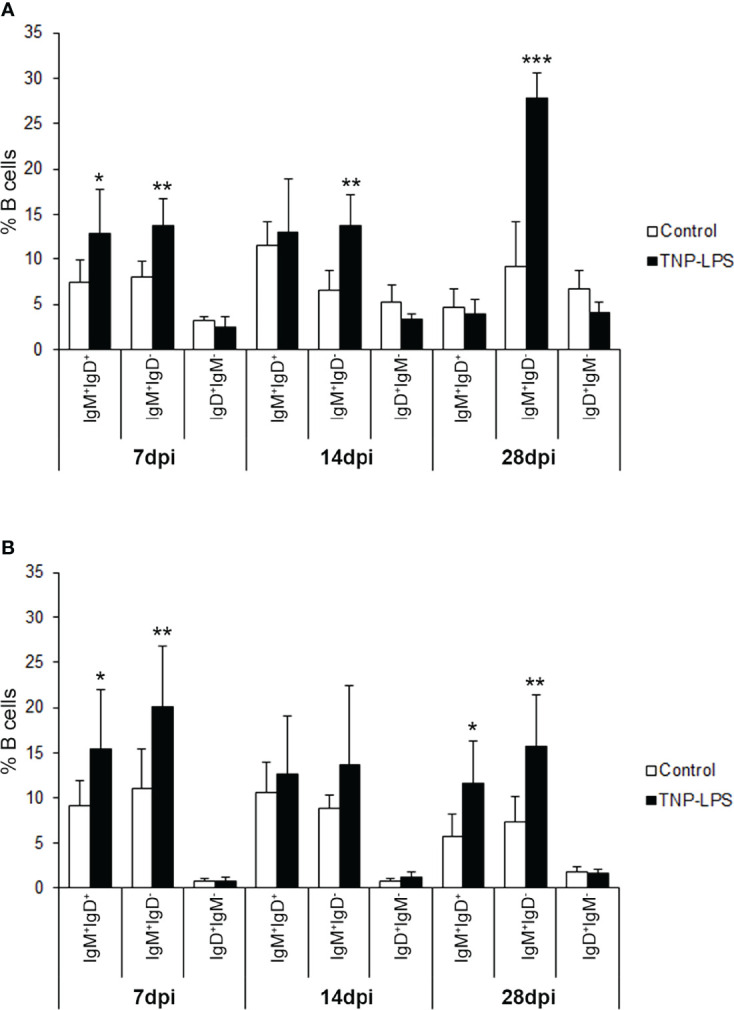
Flow cytometry analysis of the different B cell subpopulations found in the AT and the peritoneal cavity after an intraperitoneal stimulation with TNP-LPS. Isolated AT and peritoneal leukocytes from fish immunized intraperitoneally with TNP-LPS and mock immunized (control) were incubated with an anti-trout IgM mAb conjugated to phycoerythrin (PE) and an anti-trout IgD mAb conjugated to allophycocyanin (APC) and analyzed by flow cytometry. Graphs show the mean percentage of the three different B cell subpopulations (IgM^+^IgD^+^; IgM^+^IgD^-^ and IgD^+^IgM^-^) present in the AT **(A)** and the peritoneal cavity **(B)** at the three sampling times (7, 14 and 28 days post-immunization) (mean + SD; *n* = 8). Asterisks denote significantly different values between the percentages obtained in the immunized group and those of the control group (*p ≤ 0.05 **p ≤ 0.01 and ***p ≤ 0.005).

To understand which of the B cell subsets were increasing in response to TNP-LPS, we further dissected the individual response of the different subpopulations in both the AT and the peritoneal cavity. We then established that the B cell subset that significantly increases in the AT in response to TNP-LPS corresponds mainly to IgM^+^IgD^-^ B cells, especially at day 28 post-immunization, although IgM^+^IgD^+^ B cells also significantly increased in response to the stimulus at early time points (**Figure 7A**). In the case of the peritoneal cavity, it was both the IgM^+^IgD^-^ and the IgM^+^IgD^+^ B cell subpopulations that significantly increased in response to TNP-LPS at days 7 and 28 post-immunization (**Figure 7B**).

### Immunofluorescence Analysis of AT From Intraperitoneally Immunized Fish

**Figure 8:**
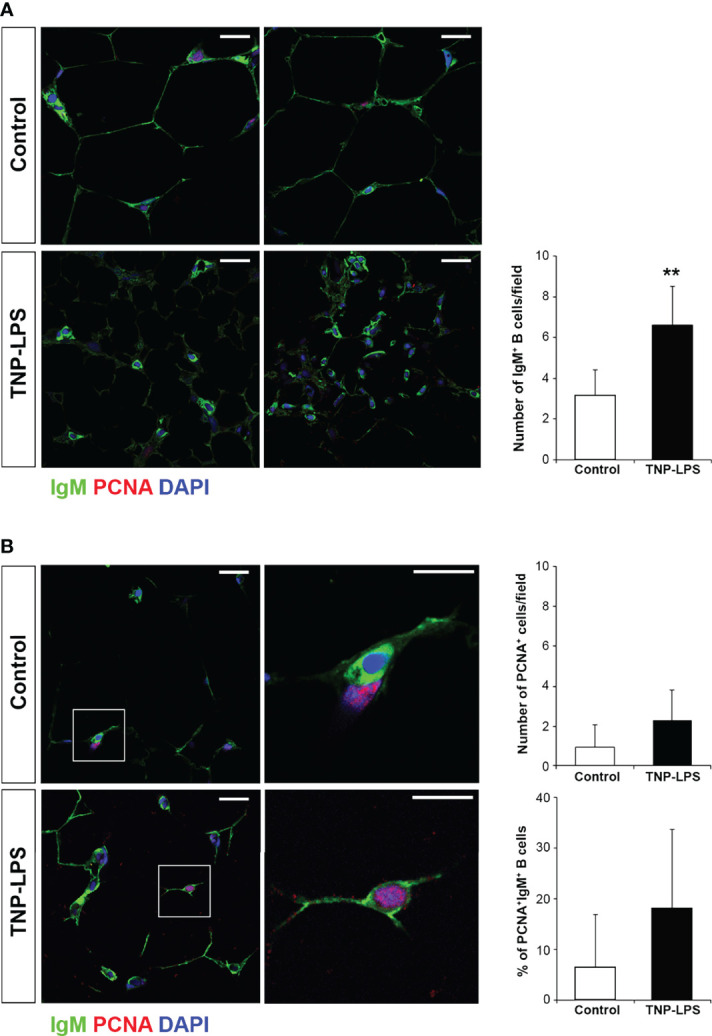
Proliferation of IgM^+^ B cells in AT cells after the intraperitoneal immunization of rainbow trout with TNP-LPS. Confocal microscopy images of rainbow trout AT sections obtained from fish intraperitoneally immunized with the antigen TNP-LPS and mock immunized (control) at day 21 post-immunization. Sections were labeled with anti-IgM (green) in combination with the anti-proliferating cell nuclear antigen (PCNA) (red). All sections were also counterstained with DAPI (blue). **(A)** Representative images for each condition are shown (scale bars = 20µm) along with a graph showing the mean number of IgM^+^ B cells in immunized fish compared to control fish, calculated in 15 digital fields (400 x magnification) from 6 different individuals (mean + SD; *n* = 6 fish). Asterisks denote significantly different values between indicated groups (**p ≤ 0.01). **(B)** Representative images for each condition are shown (scale bars = 20µm) along with higher magnification images from control and TNP-LPS immunized tissues (white square; scale bar = 5 µm). Note that in non-proliferating cells, nuclei appear blue whereas they appear violet in proliferating cells. Graphs showing the mean number of total PCNA^+^ proliferating cells as well as the mean percentage of proliferating IgM^+^/PCNA^+^ cells in immunized fish compared to those in control fish are also included, calculated in 15 digital fields (400 x magnification) from 6 different individuals (mean + SD; *n* = 6).

Having established that the percentage of IgM^+^ B cells significantly increased in the AT in response to an intraperitoneal immunization with TNP-LPS, we decided to investigate how these B cells were distributed in the tissue and whether they were proliferating in response to the antigen. For this, we used confocal microscopy, combining the anti-IgM antibody with an antibody directed against PCNA. Through this methodology, we found that, after 21 days of immunization, the number of IgM^+^ B cells increased in the interstitial spaces of the AT in immunized fish compared to control fish (**Figure 8A**), confirming our flow cytometry results. Interestingly, as a result, the size of the adipocytes seemed to decrease in immunized fish (**Figure 7A**). Nevertheless, the percentage of proliferating IgM^+^PCNA^+^ B cells in the AT of immunized fish only slightly increased compared to control fish (**Figure 8B**), suggesting that the increased number of IgM^+^ B cells observed in the AT of immunized fish is not exclusively a result of local proliferation.

## Discussion

In mammals, macrophages constitute the major leukocyte population found in the AT, although other immune cells, such as B cells, are also present, playing important roles in the maintenance of tissue homeostasis [recently reviewed in (5)]. Previous results from our lab have shown that, in rainbow trout, B cells are numerous in the AT (14). However, an extensive characterization of the different B cell subsets present in this tissue was not undertaken. In the present study, we have thoroughly characterized the B cell populations present in AT for the first time in teleosts. Initially, we phenotypically characterized these B cell subsets in homeostasis, comparing them with peritoneal and blood B cells, to then establish how they respond to an intraperitoneal immunization. At a transcriptional level, IgT expression was much lower than that of IgM in the AT (14), so, in the current work, we focused on further characterizing the non-IgT B cell subsets (B cells expressing IgM and/or IgD on the cell surface). As observed in other rainbow trout tissues, three distinct B cell subsets can be distinguished based on the levels of surface IgM and IgD expression. Thus, in the AT, as in the peritoneal cavity or blood, IgM^+^IgD^+^, IgM^+^IgD^-^ and IgD^+^IgM^-^ B cells could be identified. Nevertheless, the relative importance of each of these B cell subsets was very different in the AT when compared to the peritoneal cavity or blood. Thus, AT B cells are mostly IgM^+^IgD^-^ B cells, whereas the most abundant subpopulation of B cells in the peritoneal cavity and blood are IgM^+^IgD^+^ B cells.

In fish, as in mammals, IgM^+^IgD^+^ B cells are considered naïve B cells that upon antigen encounter, start a differentiation process towards plasmablasts and eventually plasma cells (37–39). Remarkably, AT IgM^+^IgD^+^ B cells have certain specific traits when compared to similar subsets in other tissues. Thus, IgM^+^IgD^+^ AT B cells express the highest levels of both IgD and MHC II on their cell surface, whilst being smaller than their counterparts in peritoneal cavity and blood. In mammals, B1 cells have been shown to be numerous in the AT (2, 5). Interestingly, studies from our laboratory and some others have highlighted the phenotypic and functional resemblance between fish IgM^+^ B cells and mammalian B1 cells (24, 40–43). However, the IgM^+^IgD^+^ AT B cells identified in this study have very high surface IgD levels while one of the defining characteristics of mammalian B1 cells is a low IgD surface expression (8). In any case, the marked differences in both IgD and MHC II surface levels will surely have an impact on the functionality of this B cell subset and on how these cells become activated, and this is something we will approach in future studies.

During the differentiation process, B cells loose surface IgD, augment their size and usually decrease the expression levels of many genes related to antigen presentation, including MHC II (44). Our results revealed that the predominant population of IgM^+^IgD^-^ B cells found in the AT has an augmented size and a transcriptional profile that corresponds to cells that have started a differentiation process towards plasmablasts/plasma cells when compared to naïve IgM^+^IgD^+^ B cells (higher levels of transcription of Blimp1, IRF4 and secreted IgM). Nonetheless, their surface MHC II expression levels were higher than those of naïve B cells and this could seem *a priori* surprising. However, studies in mammals have demonstrated that when plasma cells are differentiated in response to TI antigens MHC II expression is maintained (45). Thus, it was postulated that such plasma cells are not exclusively specialized in antibody secretion but retain roles in antigen presentation and pathogen clearance through a functional antigen presenting machinery. Likewise, when rainbow trout splenic B cells are stimulated with LPS, they initiate a differentiation process that goes along with an up-regulation of MHC II surface levels, while that induced by IL-6 implies a reduction of MHC II levels (34). On the other hand, and taking into account that the mammalian AT is a site were numerous B1 cells can be found, it should also be noted that, in fact, mammalian B1 and plasmablasts also share many features. Both cell subsets have very low or no surface IgD expression, larger size and high IgM secreting capacity and even higher levels of Blimp1 transcription (8, 46). Thus, it is difficult to differentiate between these two subpopulations in many occasions, including the current study, especially taking into account that a clear distinction between B1 and B2 subpopulations might not be evident in teleost fish. Nevertheless, what we can assert is that IgM^+^IgD^-^ B cells with a high IgM secreting capacity, a transcriptional profile of differentiated B cells and high MHC II surface levels constitute the main B cell subset in the rainbow trout AT in homeostasis.

Finally, IgD^+^IgM^-^ B cells were also found in the AT, however at low frequency. In rainbow trout gills and gut, where IgD^+^IgM^-^ B cells are prominent, recent evidence suggests that these cells correspond to B cells that have somehow differentiated to IgD-secreting plasmablasts (25), but their specific role or that of secreted IgD are still not well-defined.

After undertaking the characterization of AT B cells in homeostasis, we were prompted to study how these AT B cells respond to a peritoneal stimulation with a strong TI antigen, such as TNP-LPS. Previous studies had already demonstrated that the B cell population in the AT increased in rainbow trout upon an intraperitoneal stimulation (14, 16) and also after an oral vaccination (47), however this B cell response had not been fully characterized. Our results indicated that, upon an intraperitoneal injection with a TI antigen, the AT is one of the tissues that harbors antigen-specific IgM-secreting cells for long time periods, at levels similar to those of systemic immune tissues such as the spleen or the head kidney. As reported before (19, 48), the peritoneal cavity contained a very high frequency of B cells secreting TNP-specific IgMs, which seemed to be retained in this location for long time periods. Thus, in our study, while these cells started to decrease between days 14 and 28 post-immunization in the AT, they were still increasing at the peritoneal cavity during this time period. Interestingly, the B cell population that mostly increases in the AT as a consequence of the immunization is the IgM^+^IgD^-^ B cell subset, thus suggesting that the AT is an important site for accumulation of cells that have differentiated to plasmablasts/plasma cells and have a high IgM secreting capacity. In the peritoneal cavity, however, both IgM^+^IgD^-^ and IgM^+^IgD^+^ B cell subsets increased in similar proportions, although only at days 7 and 28 post-immunization. The reason why these increases were not visible in the peritoneal cavity at day 14 are unknown and should be further investigated. Of note, the increase in the number of total IgM^+^ B cells in the AT cannot be exclusively attributed to the local proliferation of cells, as established by IFA, thus B cells from the periphery (blood or peritoneal cavity) might also migrate to the AT upon peritoneal stimulation, as reported in mammals (49). Taking into account these results and the fact that the AT has been shown to be capable of collecting bacteria and other particulates from the peritoneal cavity (14), it seems quite relevant to include a characterization of AT immune response when studying the response to intraperitoneally delivered vaccines in fish. In this sense, an interesting study by Veenstra *et al.* revealed that the AT was a relevant tissue to study the side effects provoked by adjuvants included in intraperitoneal vaccine formulations in fish, as a relationship between AT immune parameters and the development of adjuvant-induced adhesions was established (17).

In conclusion, we have demonstrated that the rainbow trout AT harbors three subsets of B cells according to their IgM and IgD expression levels, namely IgM^+^IgD^+^, IgM^+^IgD^-^ and IgD^+^IgM^-^ B cells. These AT IgM^+^IgD^+^ B cells had higher levels of MHC II and IgD surface expression than their counterparts in other tissues. Similarly, IgM^+^IgD^-^ B cells which constitute the most numerous subset, although differentiated to a plasmablast/plasma cell profile, also express high levels of MHC II surface expression. Altogether, these results point to an important role of the AT B cells in antigen presentation and IgM secretion in response to peritoneal TI antigens. Thus, our results demonstrate that the fish AT is an immunocompetent organ that bears different B cell subsets that actively respond to immune stimulation.

## Data Availability Statement

The original contributions presented in the study are included in the article/**Supplementary Material**. Further inquiries can be directed to the corresponding author.

## Ethics Statement

The animal study was reviewed and approved by Ethics Committee from INIA (Code PROEX 002/17).

## Author Contributions

RS performed and analyzed most experiments with help from AM-M, EM, and PD-R. RS and AM-M carried out the *in vivo* challenge experiments with help from EM and PD-R. EM provided support with all flow cytometry experiments and performed the cell sorting. CT conceived the work and designed the experiments with help from PD-R. CT and RS wrote the main body of the paper with contributions from all other authors. All authors contributed to the article and approved the submitted version.

## Funding

This work was supported by the European Research Council (ERC Consolidator Grant 2016 725061 TEMUBLYM) and by the *Comunidad de Madrid* (grant 2016-T1/BIO-1672).

## Conflict of Interest

The authors declare that the research was conducted in the absence of any commercial or financial relationships that could be construed as a potential conflict of interest.

## Publisher’s Note

All claims expressed in this article are solely those of the authors and do not necessarily represent those of their affiliated organizations, or those of the publisher, the editors and the reviewers. Any product that may be evaluated in this article, or claim that may be made by its manufacturer, is not guaranteed or endorsed by the publisher.
