# Supplementary material for: Functional and Phenotypic Characterization of B Cells in the Teleost Adipose Tissue
Source: Front Immunol. 2022 May 10;13:868551. doi: 10.3389/fimmu.2022.868551 (PMC9127059; doi:10.3389/fimmu.2022.868551)
Supplement: Supplementary file 1 [file Presentation_1.pptx]

## Slide 1
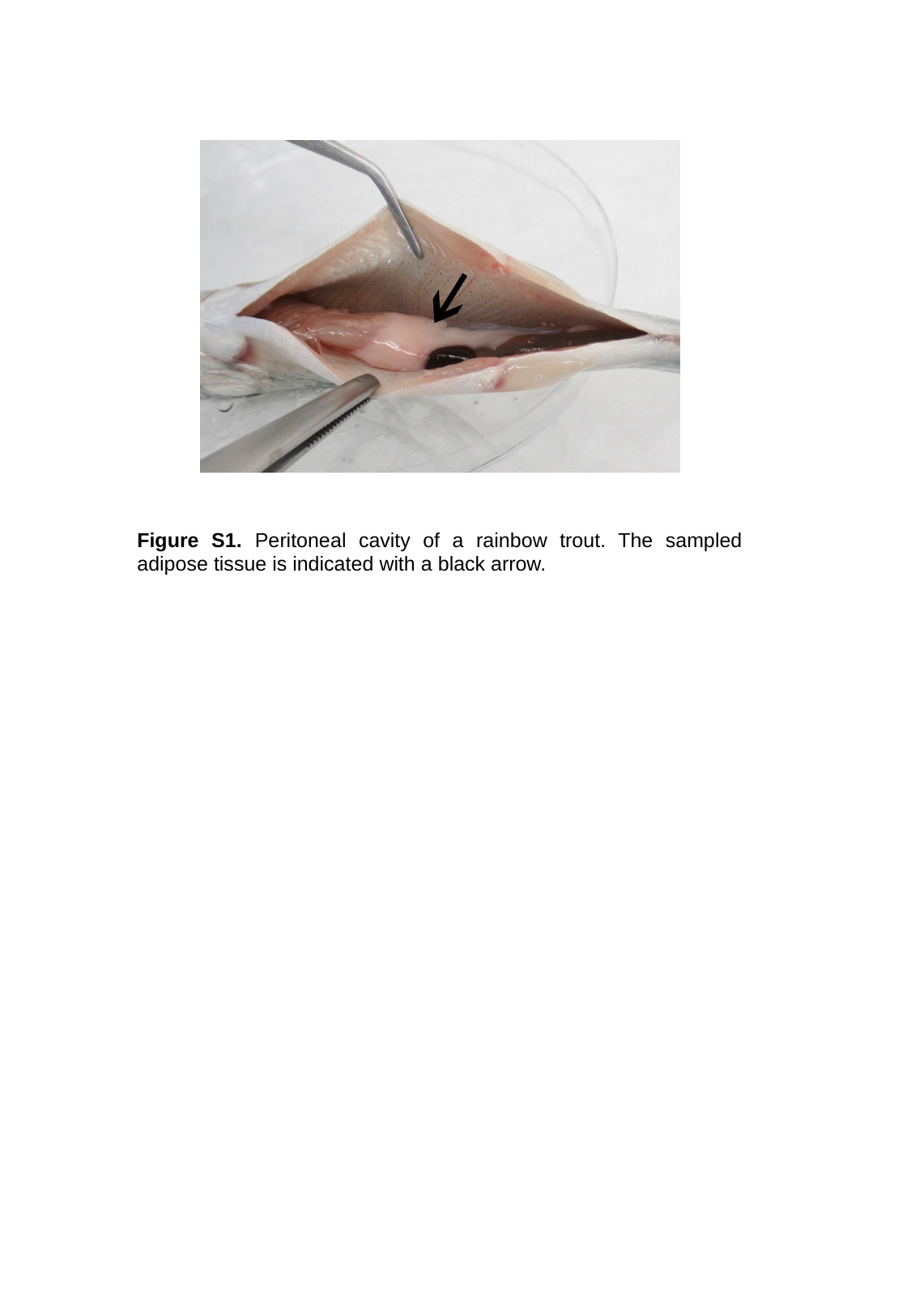

Figure S1. Peritoneal cavity of a rainbow trout. The sampled adipose tissue is indicated with a black arrow.

## Slide 2
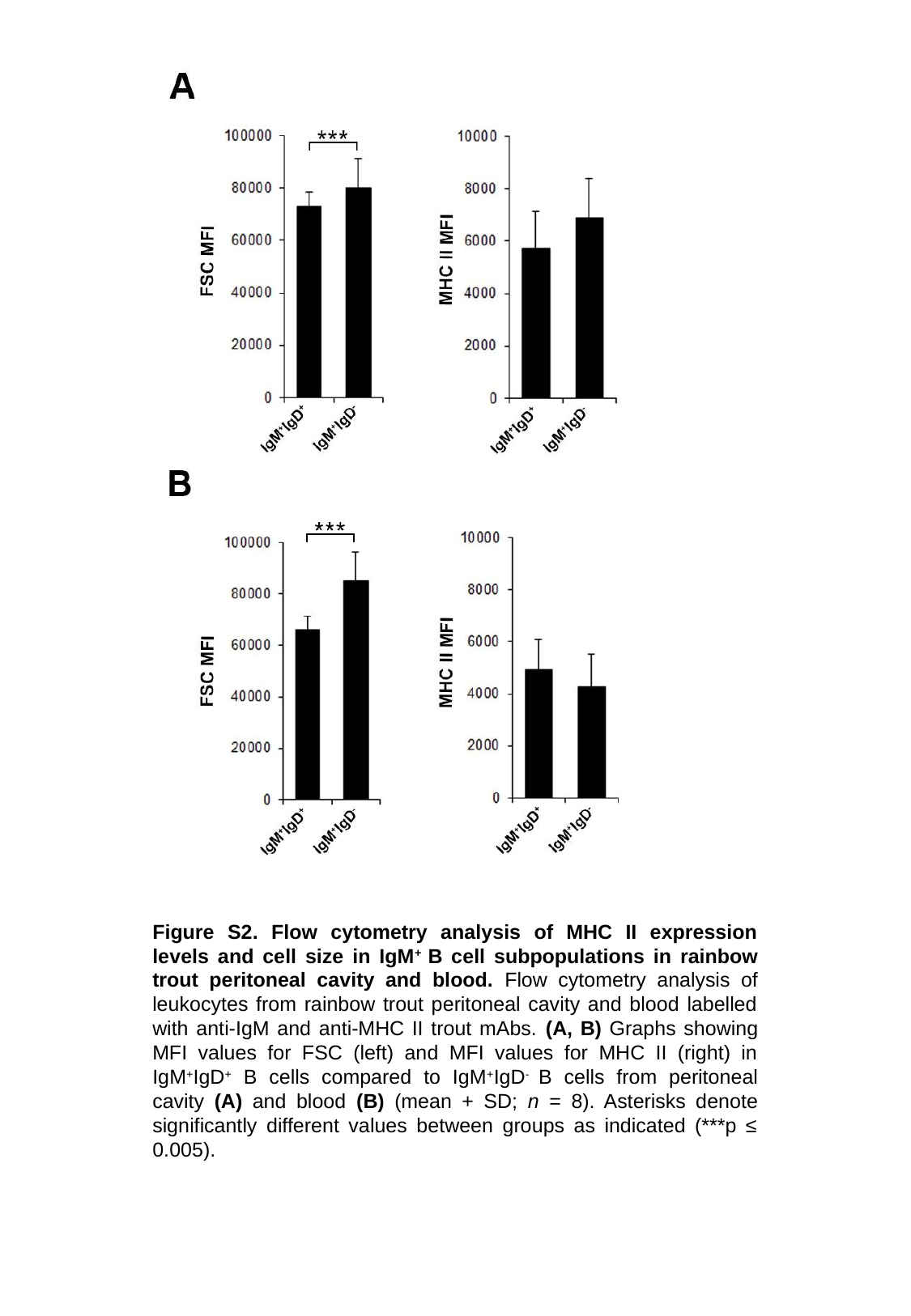

Figure S2. Flow cytometry analysis of MHC II expression levels and cell size in IgM+ B cell subpopulations in rainbow trout peritoneal cavity and blood. Flow cytometry analysis of leukocytes from rainbow trout peritoneal cavity and blood labelled with anti-IgM and anti-MHC II trout mAbs. (A, B) Graphs showing MFI values for FSC (left) and MFI values for MHC II (right) in IgM+IgD+ B cells compared to IgM+IgD- B cells from peritoneal cavity (A) and blood (B) (mean + SD; n = 8). Asterisks denote significantly different values between groups as indicated (***p ≤ 0.005).

## Slide 3
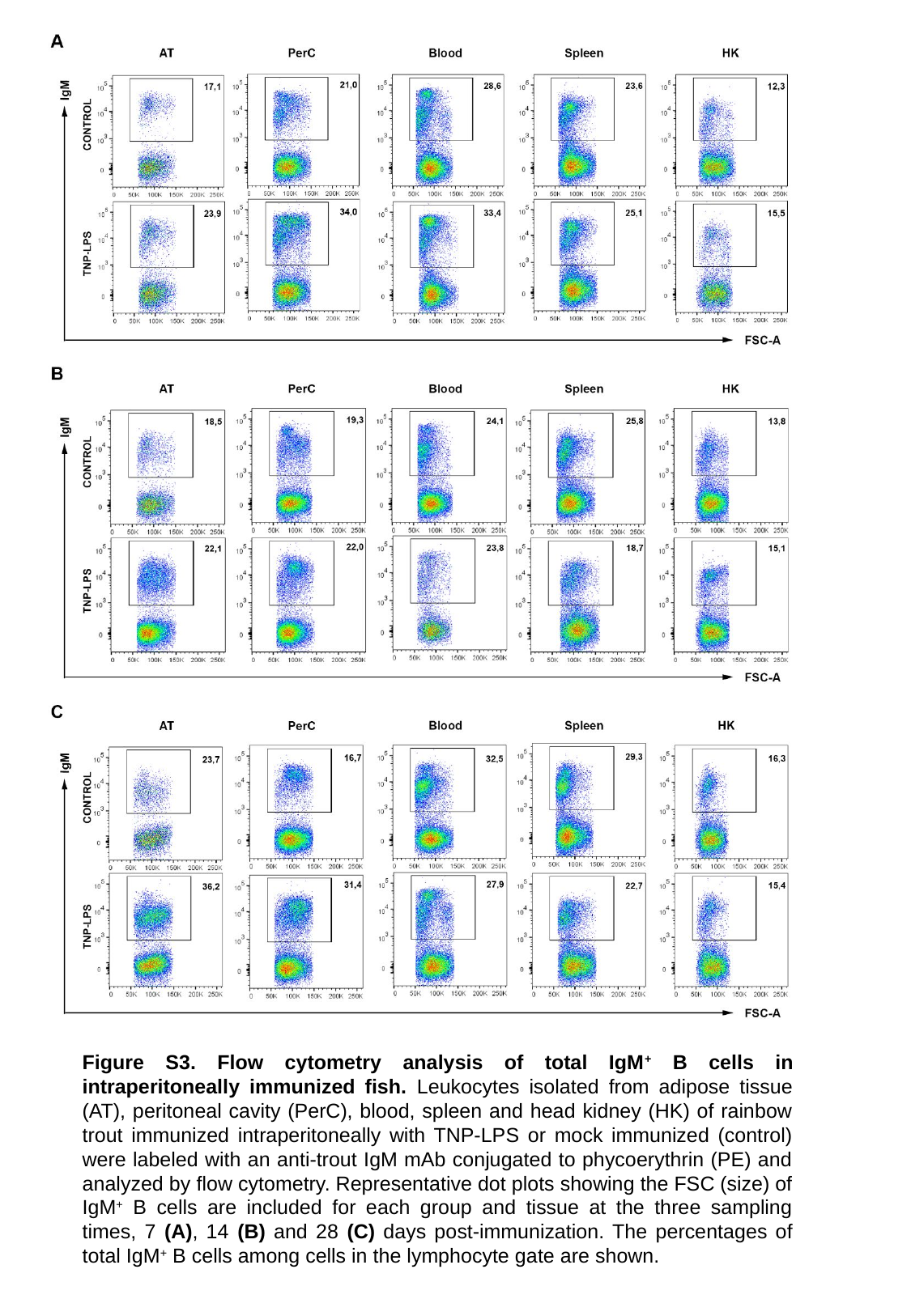

Figure S3. Flow cytometry analysis of total IgM+ B cells in intraperitoneally immunized fish. Leukocytes isolated from adipose tissue (AT), peritoneal cavity (PerC), blood, spleen and head kidney (HK) of rainbow trout immunized intraperitoneally with TNP-LPS or mock immunized (control) were labeled with an anti-trout IgM mAb conjugated to phycoerythrin (PE) and analyzed by ﬂow cytometry. Representative dot plots showing the FSC (size) of IgM+ B cells are included for each group and tissue at the three sampling times, 7 (A), 14 (B) and 28 (C) days post-immunization. The percentages of total IgM+ B cells among cells in the lymphocyte gate are shown.

## Slide 4
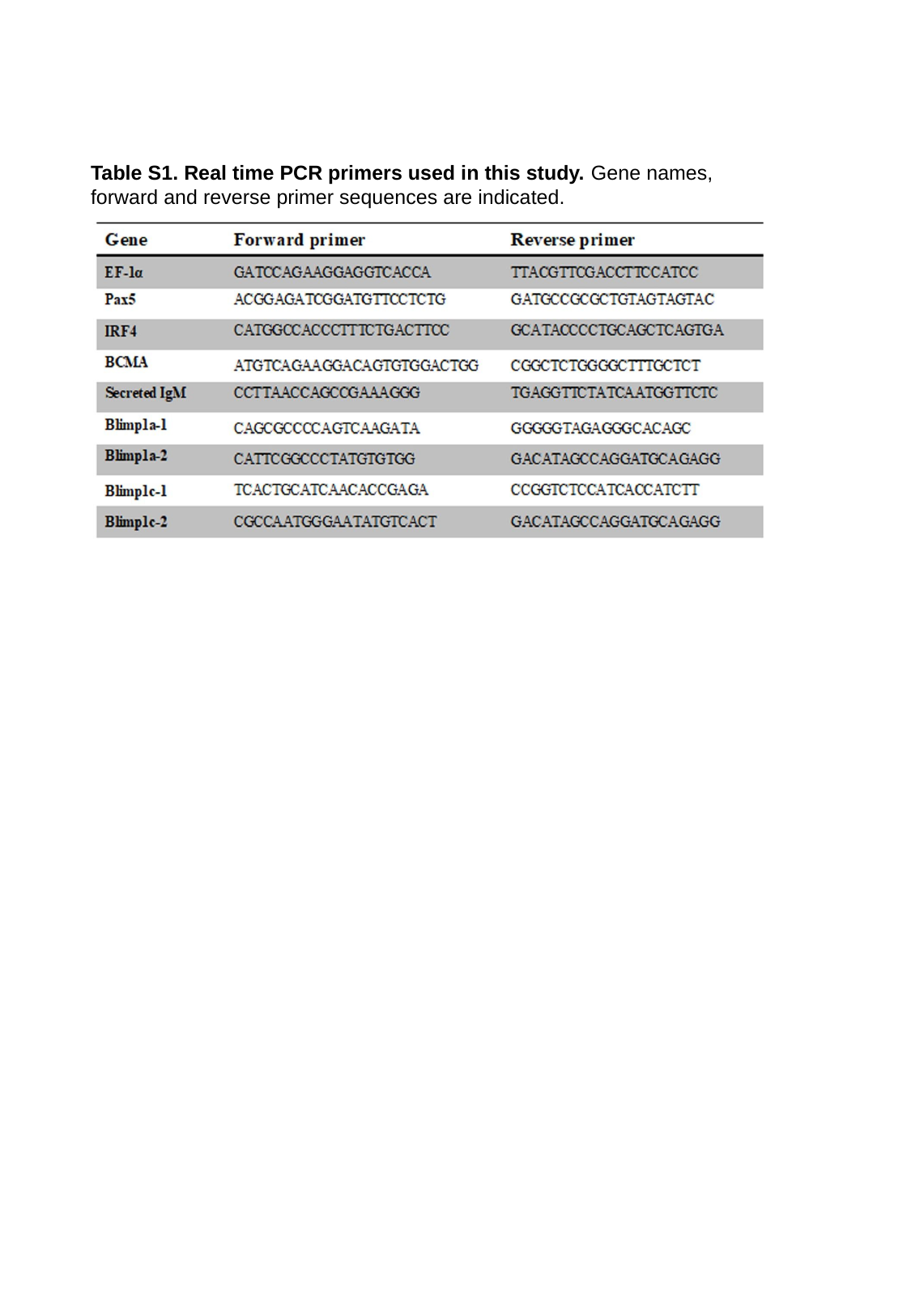

Table S1. Real time PCR primers used in this study. Gene names, forward and reverse primer sequences are indicated.
